# Association of sleep quality and psychological aspects with reports of bruxism and TMD in Brazilian dentists during the COVID-19 pandemic

**DOI:** 10.1590/1678-7757-2020-1089

**Published:** 2021-07-23

**Authors:** Karen Oliveira Peixoto, Camila Maria Bastos Machado de Resende, Erika Oliveira de Almeida, Camila Megale Almeida-Leite, Paulo César Rodrigues Conti, Gustavo Augusto Seabra Barbosa, Juliana Stuginski Barbosa

**Affiliations:** 1 Universidade Federal do Rio Grande do Norte Departamento de Odontologia NatalRN Brasil Universidade Federal do Rio Grande do Norte (UFRN), Departamento de Odontologia, Natal, RN, Brasil.; 2 Universidade Federal de Minas Gerais Instituto de Ciências Biológicas Departamento de Morfologia Belo HorizonteMG Brasil Universidade Federal de Minas Gerais, Instituto de Ciências Biológicas, Departamento de Morfologia, Belo Horizonte, MG, Brasil.; 3 Universidade de São Paulo Bauru Orofacial Pain Group BauruSP Brasil Universidade de São Paulo, Bauru Orofacial Pain Group, Bauru, SP, Brasil.; 4 Universidade de São Paulo Faculdade de Odontologia de Bauru Departamento de Prótese e Periodontia BauruSP Brasil Universidade de São Paulo, Faculdade de Odontologia de Bauru, Departamento de Prótese e Periodontia, Bauru, SP, Brasil

**Keywords:** Coronavirus infections, Psychological distress, Anxiety, Depression, Sleep disorders, Bruxism, Temporomandibular joint disorder

## Abstract

Dentists are exposed to contamination by SARS-CoV-2 due to dental interventions, leading to a state of alert and potential risk of negative impact in mental health and sleep quality, associated with Temporomandibular Disorder (TMD) and bruxism.

**Objective:**

to evaluate the psychosocial status, sleep quality, symptoms of TMD, and bruxism in Brazilian dentists (DSs) during the COVID-19 pandemic.

**Methodology:**

The sample (n=641 DSs) was divided into three groups (quarantined DSs; DSs in outpatient care; and frontline professionals), which answered an electronic form containing the TMD Pain Screening Questionnaire (Diagnostic Criteria for Temporomandibular Disorders – DC/TMD), the Pittsburgh Sleep Quality Index (PSQI), the Depression, Anxiety and Stress Scale (DASS-21), and the sleep and awake bruxism questionnaire. ANOVA test and Mann Whitney post-test were used, with Bonferroni adjustment (p<0.016) and a 95% confidence level.

**Results:**

Probable TMD was found in 24.3% (n=156) of the participants, while possible sleep and awake bruxism were diagnosed in 58% (n=372) and 53.8% (n=345) of them, respectively. Among all variables evaluated, only symptoms of depression were significantly greater in the quarantined DSs group when compared to those who were working at the clinical care (p=0.002). Working DSs were significantly less likely (OR=0.630, p=0.001) to have depressive symptoms. Those who were not worried or less worried about the pandemic were less likely to experience stress (OR=0.360), anxiety (OR=0.255), and poor sleep quality (OR=0.256). Sleep had a strong positive and moderate correlation with psychological factors on frontline workers and DSs in outpatient care, respectively.

**Conclusion:**

The results suggest confinement may have a more negative impact on the life of DSs than the act of being actively working. The concern about Covid-19 and poor sleep quality was significantly prevalent and may negatively affect the quality of life of DSs. Thus, further research on the topic is needed.

## Introduction

Unpredictable or incomprehensible life situations, as the current pandemic caused by the Coronavirus Disease 2019 (COVID-19), are important sources of stress.^1^ The causative agent of the disease is the new Coronavirus related to the Severe Acute Respiratory Syndrome (SARS-CoV-2).^2^ The virus is transmitted from person to person, mainly through direct contact or through virus-laden droplets propelled by the coughing or sneezing from an infected individual.^3^ The first case of the disease occurred in Wuhan, China, in December 2019 and spread rapidly around the world, being classified in March 2020, by the World Health Organization (WHO), as a pandemic of great risk to international health.^4^

To reduce the transmission of Covid-19, broad public health measures have been adopted, such as social isolation and quarantine.^2^ Confinement can be stressful,^5^ bringing with it challenges of considerable impact in the daily routine of individuals, such as reducing social interaction, working long hours under new circumstances, dealing with the fear of contamination, and constantly worrying about preventive measures.^6^ The quarantine substantially increases anxiety and may negatively affect general health, leading to other health problems.^7^ Moreover, possible financial difficulties from being away from work contribute to anxiety, uncertainty, and stress.^6^

Health-care workers in all areas worldwide are facing a major challenge, as they are particularly exposed to various stressors during the pandemic.^4^ The ones who are in quarantine need to reconcile their domestic responsibilities with studies or work, often under more stressful conditions due to family life, especially for those with children.^6^ Those who remain at work, however, need to deal with the fear of infecting themselves or their families, which leads to a permanent state of vigilance, and even exhaustion from wearing personal protective equipment (PPE).^4^ Those on the frontline in hospitals face the need to use even more equipment, as they frequently deal with infected patients, besides the increased workload and constant work pressure.^8^

During the SARS-CoV outbreak in 2003, most health-care workers experienced high levels of symptoms of stress, anxiety, and depression.^9^^,^^10^ Despite protective measures, professionals were infected with SARS during aerosol-generating procedures.^11^ Hence, dental professionals who are strongly exposed to it during dental procedures may be quite susceptible to SARS-CoV-2 contamination due to clinical practice.^12^

Moreover, it has been shown, among health-care professionals, dentistry has one of the highest levels of stress load and risk of developing musculoskeletal disorders, such as temporomandibular disorders (TMD).^13^^,^^14^ TMD is defined as a series of clinical problems involving the masticatory muscles, the temporomandibular joint (TMJ), and associated structures.^15^ It is one of the musculoskeletal conditions that mostly results in pain and disability, and the most frequent condition of chronic orofacial pain.^16^ Psychosocial factors and sleep disorders are known to contribute to the perpetuation of TMD pain^17^ and some studies demonstrate a positive association between TMD and bruxism.^18^^,^^19^ Also, psychosocial factors were related to increased masticatory muscle activity in awake bruxism.^20^ Furthermore, stress and anxiety can also affect the quality of life and sleep of individuals.^1^^,^^21^

Therefore, this study aims to verify sleep quality, psychological aspects, reports of bruxism, and TMD symptoms in Brazilian dentists (DSs) who were on the frontline in hospitals, in outpatient care, and quarantined at home during the COVID-19 pandemic. The first hypothesis was that dentists who were on the frontline would have a high prevalence of sleep disorders, psychological changes, TMD symptoms, and bruxism (H1). The second hypothesis was the existence of an association between concerns regarding the pandemic and their working status with psychosocial aspects, sleep quality, TMD symptoms, and bruxism (H2). The third hypothesis was the existence of a positive correlation between sleep quality and psychosocial aspects in different groups (H3).

## Methodology

This is an observational cross-sectional study approved by the Central Ethics Committee on Research from the Universidade Federal do Rio Grande do Norte (UFRN) (CAAE 4.062.172), developed according to the norms and requirements of resolution 466/2012 from the Brazilian regulatory agency of ethics in research with human beings.

The research took place in a single moment through an electronic form developed on the Google Forms platform that was filled out by DSs registered in the Federal Council of Dentistry (CFO) from May 2020 to June 2020. The professionals were invited to participate in the survey by electronic messages containing the link to the form via direct contact from the social networks of the researchers. The convenience sample consisted of 641 DSs, calculated from the 348,703 professionals enrolled in the CFO until May 2020, with an error of 5% and a confidence level of 99%.

Participants were allocated into three groups according to their working status during the COVID-19 pandemic. Group 1 (G1): professionals who were at home in quarantine; Group 2 (G2): professionals performing only outpatient care (in public or private offices); and Group 3 (G3): professionals working on the frontline in hospitals.

Initially, patients answered the sociodemographic questionnaire (Appendix I). The degree of concern about the pandemic was measured on a Likert Scale and categorized as not worried, little worried, worried, or extremely worried. TMD Pain Screening from the Diagnostic Criteria for Temporomandibular Disorders (DC/TMD) in its Brazilian-Portuguese version was used.^22^ It contains three questions related to the last 30 days: the first about the duration of pain in the temporal or mandibular region, the second about jaw pain or stiffness upon waking, and the third about activities that modify jaw or temporal pain. From their answers, subjects were categorized as with symptoms of TMD and without symptoms of TMD.

The Depression, Anxiety, and Stress Scale (DASS-21) in the Brazilian-Portuguese version^23^ was used simultaneously to measure and distinguish depression, anxiety, and stress symptoms. Its items are divided into three factors (depression items: 3, 5, 10, 13, 16, 17, 21; anxiety items: 2, 4, 7, 9, 15, 19, 20; and stress items: 1, 6, 8, 11, 12, 14, 18). The responses to them range from 0 (does not apply to me at all) to 3 (applies to me very much or most of the time).

For sleep quality, the Brazilian-Portuguese version of the Pittsburgh Sleep Quality Index (PSQI)^24^ was used. It analyzes the subjective quality of sleep, both quantitatively and qualitatively, in the past 30 days. It consists of 19 questions divided into seven components (subjective sleep quality, sleep latency, sleep duration, habitual sleep efficiency, sleep disorders, use of sleep medications, daytime dysfunction) with scores from 0 to 3. An overall score of 0 to 21 is obtained from the sum of the components and higher scores are indicative of worse sleep quality. Overall score greater than 5 was categorized as a poor sleeper and lower than 5 as a good sleeper.

The evaluation of bruxism was performed through self-reports, assessed through the frequency of the behavior in the last month related to some parafunctional and occupational habits during sleep and upon waking (sleep bruxism), or during the day (awake bruxism).^25^ The following questions were extracted from the Oral Behavior Checklist (OBC):^25^ Clenching or grinding teeth when asleep, based on any information you may have? Sleeping in a position that puts pressure on the jaw (for example, on the stomach, on the side)?; Grinding teeth together during waking hours? Clenching teeth together during waking hours? Pressing, touching, or holding teeth together other than while eating (that is, contact between upper and lower teeth)? Holding, tightening, or tensing muscles without clenching or bringing teeth together? Placing tongue between teeth? Biting, chewing or playing with your tongue, cheeks, or lips.

### Statistical analysis

The statistical analysis was performed using the software Statistical Package for the Social Science (SPSS) version 22.0. The descriptive analysis was performed with absolute values, frequencies, and measures of central tendency and variability. Pearson's chi-square test was used to verify the association between the dependent categorical variables (anxiety, stress, depression, TMD screening, sleep, and awake and sleep bruxism) with the examined groups (G1, G2, and G3; their professional performance during the pandemic). For comparing symptoms of anxiety, depression, stress, and sleep quality, the One-way ANOVA test was used, followed by the Mann-Whitney post-test, with the Bonferroni adjustment <0.016 (three comparisons). Spearman's correlation was performed between sleep quality and psychological variables (symptoms of anxiety, stress, and depression). Binary logistic regression was also used between dependent variables (symptoms of anxiety, stress, depression, TMD screening, subjective quality of sleep, possible awake and sleep bruxism) and its relationship with the variables representing the pandemic (their specific working status during the pandemic and concerns about it), to analyze their influence in the presence of the evaluated conditions. Also, those that were not directly related to the variables associated with the pandemic were placed in another analysis, but as dependent variables, along with the ones showing a significant result, to verify the presence of a possible indirect relationship.

## Results

The sample of this study was composed of 641 dentists (age 39 years old ± 10.56, 74% female, 61.2% married) who answered the online questionnaire. The exclusion of some individuals who sent the form in duplicate caused a loss of 22 questionnaires (3% of the sample). Among the participants, 208 were quarantined (G1), 401 in outpatient care (G2), and 32 on the frontline in hospitals (G3). There was no statistically significant difference regarding gender among groups (p=0.083) (Table 1).

**Table 1:** Distribution regarding the variables gender, marital status and concerns about pandemic among the 3 different groups of dentists. Absolute number (n), relative (%) and statistical significance (p)

Gender	G1 (n=206)	G2 (n=400)	G3 (n=32)	Total (n=638)	p*
Male	20.9% (n=43)	28.5% (n=114)	18.8% (n=6)	25.5% (n=163)	0.72
Female	79.1% (n=163)	71.5% (n=286)	81.3% (n=26)	74.5% (n=475)	
**Marital Status**	**G1 (n=206)**	**G2 (n=398)**	**G3 (n=32)**	**Total (n=636)**	**p**
Married/Common-law	60.2% (n=124)	61.6% (n=245)	62.5% (n=20)	61.2% (n=389)	0.49
Single	33.5% (n=69)	30.9% (n=123)	37.5% (n=12)	32.1% (n=204)	
Divorced/Widow	6.3% (n=13)	7.6% (n=30)	0.0% (n=0)	6.8% (n=43)	
**Concerns about COVID-19**	**G1 (n=208)**	**G2 (n=400)**	**G3 (n=32)**	**Total (n=640)**	p*
Not worried or little worried	4.3% (n=9)	9.3% (n=37)	9.4% (n=3)	7.7% (n=49)	0.89
Worried to extremely worried	95.7% (n=199)	90.8% (n=363)	90.6% (n=12)	92.3% (n=591)	

* Pearson's chi-square test. G1: quarantined. G2: outpatient care/practice. G3: frontline in hospitals.

When asked about the level of concern regarding the current pandemic, 92.3% of the respondents were moderate to extremely worried (Table 1). TMD symptoms were seen in 24.3% (n=156) of them, with no significant difference among groups (p=0.86) (Table 2). The self-reported bruxism in the sample was 58.03% for sleep bruxism (SB) and 53.82% for awake bruxism (AB), with no considerable difference among groups (p=0.33, p=0.79, respectively). Regarding sleep, 93.4% of the participants had a score above 5 in the PSQI questionnaire, which could be classified as poor sleep quality with no difference among them (p=0.72) (Table 2). The PSQI mean score and standard deviation for the groups were 9.60 (3.09), 9.51 (2.98), and 9.13 (2.68) for quarantined (G1), outpatient care (G2), and frontline in hospitals (G3), respectively.

**Table 2:** Descriptive table of variables analyzed among groups of dentists according to the applied questionnaires. Absolute number (n), relative (%) and statistical significance (p)

Reporting Sleep Bruxism	G1 (n=208)	G2 (n=401)	G3 (n=32)	p*
Without SB	39.4% (n=82)	42.4% (n=170)	53.1% (n=17)	0.33
Possible SB	60.6% (n=126)	57.6% (n=231)	46.9% (n=15)	
**Reporting Awake Bruxism**	**G1 (n=208)**	**G2 (n=401)**	**G3 (n=32)**	p*
Without AB	47.1% (n=98)	46.1% (n=185)	40.6% (n=13)	0.79
Possible AB	52.9% (n=110)	53.9% (n=216)	59.4% (n=19)	
**TMD Pain Screening**	**G1 (n=208)**	**G2 (n=401)**	**G3 (n=32)**	p*
Without symptoms of TMD	75.5% (n=157)	76.1% (n=305)	71.9% (n=23)	0.86
With symptoms of TMD	24.5% (n=51)	23.9% (n=96)	28.1% (n=9)	
**PSQI > 5**	**G1 (n=208)**	**G2 (n=401)**	**G3 (n=32)**	p*
Good sleeper	7.7% (n=16)	6.0% (n=24)	6.3% (n=2)	0.72
Poor sleeper	92.3% (n=192)	94.0% (n=377)	93.8% (n=30)	

* Pearson chi-square test with significance level <0.05; sleep bruxism (SB); awake bruxism (AB); temporomandibular disorder (TMD); quarantined (G1); outpatient care/practice (G2); frontline in hospitals (G3).

Stress symptoms had a similar distribution among the studied groups, with no significant difference (p=0.127), as well as anxiety (p=0.208). For depressive symptoms, those who were quarantined had more symptoms, but the significant difference occurred only with the outpatient/office care group (p=0.002; Bonferroni adjustment p<0.016) (Table 3).

**Table 3:** Comparison of psychosocial factors obtained by the Depression, Anxiety and Stress Scale (DASS-21) among groups during the COVID-19 pandemic. Average, standard deviation (SD), confidence interval (CI), lower limit (LL) and upper limit (UL) minimum and maximum, and statistical significance (p)

DASS-21					
**Stress symptoms**					
**Group**	**Average (SD)**	**CI 95%**		**Minimum**	**Maximum**
		LL	L		
G1 (n=208)	13.35 (7.77)	12.28	14.41	0	40
G2 (n=401)	12.07 (7.66)	11.32	12.82	0	40
G3 (n=32)	13.38 (7.86)	10.54	16.21	0	38
Total (n=641)	12.55 (7.72)	11.95	13.15	0	40
p*	0.127				
**Anxiety symptoms**					
**Group**	**Average (SD)**	**CI 95%**		**Minimum**	**Maximum**
		LL	UL		
G1 (n=208)	6.39 (6.13)	5.56	7.23	0	32
G2 (n=401)	5.50 (5.94)	4.91	6.08	0	34
G3 (n=32)	5.56 (5.09)	3.72	7.40	0	18
Total (n=641)	5.79 (5.97)	5.33	6.25	0	34
p*	0.208				
**Depression symptoms**					
**Group**	**Average (SD)**	**CI 95%**		**Minimum**	**Maximum**
		LL	UL		
G1 (n=208) $	8.07 (6.85)	7.13	9.00	0	34
G2 (n=401) $	6.62 (6.87)	5.94	7.29	0	42
G3 (n=32)	7.25 (7.01)	4.72	9.78	0	28
Total (n=641)	7.12 (6.89)	6.59	7.66	0	42
p*	0.048				

* Oneway ANOVA $ Mann Whitney (groups in which there was a statistically significant difference among them).

G1: quarantined. G2: outpatient care/practice. G3: frontline in hospitals. Significance level p (<0.05).

There was also a significant correlation between sleep and psychological factors. In the quarantined group, sleep quality demonstrated a moderate correlation with stress, anxiety, and depression (Spearman's Rank Correlation Coefficient of 0.431, 0.406, 0.404, respectively; p<0.001). For those who were working with outpatient care, there was a moderate correlation between sleep quality and stress, and with anxiety, as well as a weak association between sleep quality and depression symptoms (Spearman's Rank Correlation Coefficient of 0.404, 0.403, 0.376 respectively, p<0.001). For the frontline group, there was a strong correlation between sleep quality and stress, anxiety, and depression symptoms (Spearman's Rank Correlation Coefficient of 0.592, 0.739, 0.686 respectively, p<0.001).

To find relations between the dependent variables (anxiety, stress, depression, TMD screening, subjective sleep quality, possible sleep, and awake bruxism) and independent variables representing the pandemic (specific working status during the pandemic and concerns about it), the first block of models was designed (Table 4). It was noticed that DSs who were actively working were significantly (p=0.001) less likely (OR=0.630) to have symptoms of depression than those who were quarantined.

**Table 4:** Logistic regression models for anxiety, depression, stress, and sleep quality according to variables that measured the characteristics from the pandemic (working status during pandemic and concerns about it)

MODEL	INDEPENDENT VARIABLES	p**	OR	CI (95%)
Depression	Working status during the pandemic	0.010	0.630	0.443-0.897
	Concern about the pandemic*	0.083	0.517	0.245-1.091
Stress	Working status during the pandemic	0,956	0.991	0.727-1.351
	Concern about the pandemic*	0.014	0.360	0.159-0.816
Anxiety	Working status during the pandemic	0,321	0.838	0.591-0.626
	Concern about the pandemic*	0.002	0.255	0.107-0.609
Sleep Quality	Working status during the pandemic	0.45	0.879	0.628-1.229
	Concern about the pandemic*	<0.001	0.256	0.122-0.539

** p: p-value. OR: Odds Ratio. CI: Confidence Interval.

* Inversion of the variable categorization by model.

It was also observed individuals who declared they were not worried or little worried about the pandemic had a lower chance of showing symptoms of stress (OR=0.360), anxiety (OR=0.255), and poor sleep (OR=0.256) in a significant way (p=0.014, p=0.002, p<0.001, respectively). TMD pain symptoms, self-reported awake, and sleep bruxism were directly associated with the depression, stress, anxiety symptoms, and sleep quality variables. They were also indirectly related to the pandemic variables (specific working status during the pandemic and concerns about it), either as a protective factor (in absence of the symptoms, there was a reduction in the number of chances of presenting the assessed condition) or a risk factor (the presence of the element increases the chances of the pathology) (Table 5).

**Table 5:** Logistic regression models for TMD, awake and sleep bruxism according to psychological variables and sleep quality

MODEL	INDEPENDENT VARIABLES (pandemic)	p	OR	CI (95%)
TMD Screening	Depression*	<0.001	0.425	0.292-0.618
	Stress*	<0.001	0.147	0.096-0.226
	Anxiety*	<0.001	0.287	0.198-0.418
	Sleep Quality	<0.001	3.010	2.064-4.389
Possible Awake Bruxism	Depression*	<0.001	0.434	0.30-0.617
	Stress*	<0.001	0.350	0.244-0.503
	Anxiety*	<0.001	0.318	0.224-0.453
	Sleep Quality	<0.001	1.68	1.230-2.312
Possible Sleep Bruxism	Depression	0.024	1.491	1.055-2.039
	Stress	0.001	1.777	1.248-2.531
	Anxiety*	<0.001	0.496	0.351-0.701
	Sleep Quality	<0.001	1.793	1.301-1.165

B: beta, p: p-value, OR: Odds Ratio, CI: Confidence Interval,

* protection factor.

Table 5 demonstrates individuals without symptoms of depression, anxiety, and stress are less likely to have TMD pain symptoms and less likely to report awake bruxism (p<0.001). Individuals with worse subjective sleep quality were more likely to have TMD pain symptoms (p<0.001, OR=3.01), report awake bruxism (p<0.001, OR=1.68), and sleep bruxism (p<0.001, OR=1.79).

## Discussion

This study evaluated the psychosocial status, sleep quality, TMD symptoms, and self-reported bruxism in dentists according to three working statuses during the COVID-19 pandemic, namely quarantined, working in outpatient care, or on the frontline in hospitals. Among all variables evaluated, only symptoms of depression were significantly greater in the quarantined group in comparison to those who were working at outpatient care. DSs that were working are significantly less likely to have depressive symptoms and those who were less concerned about the pandemic were less likely to experience stress anxiety and poor sleep quality. Sleep had a strong positive correlation with psychological factors in frontline workers and moderate for outpatient care professionals.

The hypothesis H1 was not confirmed, once frontline professionals did not show a higher prevalence of the studied variables in comparison to other groups. However, hypothesis H2 was confirmed, as there was a relationship between the variables representing the pandemic and the studied variables. Hypothesis H3 confirmed the existence of a positive correlation between sleep quality and psychosocial aspects in the observed groups.

A high number of participants were worried about the pandemic, representing more than 90% of the sample. This concern is not uncommon in the current moment, once most people have undergone major changes in their routines, dealing with insecurities about their health, duration of the pandemic, and financial situation, in face of this new reality.^6^

The COVID-19 pandemic affects both the physical and mental health of individuals,^9^^,^^26^^,^^27^ with health-care workers being particularly subjected to various stressors, which may lead to higher levels of emotional distress.^4^ Shacham, et al.^28^ (2020) found an elevated risk of psychological distress in 11.5% (n=39) of the dentists evaluated in Israel during the COVID-19 pandemic. Huang, et al.^29^ (2020) demonstrated symptoms of anxiety and stress were present in 23.04% and 27.39% of 230 participants (70 doctors and 160 nurses), respectively, in a survey conducted in an infectious disease hospital for COVID-19 in China. Such results were similar to those found in the current study regarding symptoms of stress (30.3%, n=194) and anxiety (33.7%, n=216).

Depression symptoms were more frequent in dentists that were isolated in quarantine. Brooks, et al.^30^ (2020) report quarantine can have a considerable impact on the mental health of the population. This fact may be related to the reduction of income and the decline in social relationships. Income uncertainty can contribute to psychological changes in individuals who are out of work as they begin to question their financial future. According to the United Nations, social isolation can be aggravated by the suffering caused by the loss of income and jobs (https://bit.ly/2XsafSl).^31^ The quarantine can also cause greater stress due to the inability to engage in rewarding activities,^6^ and the decrease in social relationships, which may exacerbate feelings of loneliness, being an important predictor for depression.^32^ Moreover, the psychological impact of the quarantine is broad, and it can be long-lasting, with long-term consequences,^30^ associated with psychological symptoms.^33^

Some studies indicate frontline professionals are more likely to present psychological changes.^9^^,^^34^^,^^35^ Nevertheless, in this study, there was no significant difference among groups for anxiety and stress symptoms. It is important to highlight the number of dentists working in the frontline participating in our study (n=32) may have been insufficient to verify a higher prevalence of psychological symptoms. This might be the result of a smaller number of those professionals working in hospitals, or even those on the frontline that have shown no interest or time to participate in the research.

More than 90% of the assessed dentists showed poor sleep quality. This fact may be related to anxiety due to the current situation and negative effects on factors that influence sleep quality.^6^^,^^8^ It is also possible increased use of social media during the pandemic may impair sleep quality, as screen exposure close to bedtime can negatively impact sleep.^6^ This result deserves attention once sleep quality is an important health indicator and may even help to strengthen the immune system against infections.^36^

Furthermore, sleep is also an important factor in regulating behavior and emotions, and sleep disorders can have direct consequences on the following day, especially in emotional functioning.^37^ Moreover, poor sleep quality is often related to psychological factors, such as stress and anxiety.^38^ Liu, et al.^35^ (2020) evaluated sleep and emotional aspects in health-care workers in China and observed 50.7%, 44.7%, 73.4%, and 36.1% of 1,563 professionals reported depressive symptoms, anxiety, stress, and sleep disorders, respectively. Here, sleep quality was significantly correlated with stress, anxiety, and depressive symptoms, especially in the frontline group, which strengthens the relationship between sleep and emotions.

Unpredictable or incomprehensible life situations, as the current pandemic caused by the Coronavirus Disease 2019 (COVID-19), are important sources of stress.^1^ The causative agent of the disease is the new Coronavirus related to the Severe Acute Respiratory Syndrome (SARS-CoV-2).^2^ The virus is transmitted from person to person, mainly through direct contact or through virus-laden droplets propelled by the coughing or sneezing from an infected individual.^3^ The first case of the disease occurred in Wuhan, China, in December 2019 and spread rapidly around the world, being classified in March 2020, by the World Health Organization (WHO), as a pandemic of great risk to international health.^4^ To reduce transmission, extensive public health measures have been adopted, such as social distancing.^2^ However, mass confinement can be stressful^5^ and quarantine substantially increases anxiety and may negatively impact general health leading to other health problems.^6^

To reduce the transmission of Covid-19, broad public health measures have been adopted, such as social isolation and quarantine.^2^ Confinement can be stressful,^5^ bringing with it challenges of considerable impact in the daily routine of individuals, such as reducing social interaction, working long hours under new circumstances, dealing with the fear of contamination, and constantly worrying about preventive measures.^6^ The quarantine substantially increases anxiety and may negatively affect general health, leading to other health problems.^7^

In contrast, the statistical analysis highlighted participants without symptoms of depression, anxiety, and stress were less likely to present TMD symptoms, which corroborates studies that relate it to the presence of higher levels of stress, anxiety, and depression.^41^^,^^42^ A three-year-long cohort study showed depression, perceived stress, and mood were predictors of a risk two to three times greater of having TMD (p<0,05).^42^ Another study found the presence of emotional overload determined a higher frequency of TMD and oral parafunctions.^43^

Anxiety, associated with a stressful event, such as the current pandemic, can exacerbate bruxism.^44^ Participants without symptoms of depression, anxiety, and stress were less likely to report awake bruxism, which agrees with previous studies that demonstrate the relationship between awake bruxism and psychosocial factors.^45^ Patients with high levels of stress may be almost six times more likely to report awake bruxism.^46^^,^^47^

Moreover, dentists with poor sleep quality were statistically related to a greater chance of awake (p<0.001, OR=1.68) and sleep bruxism (p<0.001, OR= 1,79). Ahlberg, et al.^48^ (2008) noticed a relationship between bruxism and sleep disorders, being significantly related to frequent awakenings. Although not verified in this study, it is important to note the reporting of sleep bruxism may be associated with sleep disorders such as obstructive sleep apnea syndrome and insomnia.^49^ Awake bruxism is strongly influenced by psychological factors,^20^ which demonstrated a positive association with sleep quality in this study and may indirectly explain the relationship between sleep quality and awake bruxism.

This study has some limitations. First, it was not possible to compare results with those of the general population, nor to distinguish pre-existing and new symptoms. Moreover, there might be bias related to the small sample of dentists working on the frontline.

Thus, based on the data presented and limitations of the study, it is essential to further research this topic, especially in a post-pandemic scenario, once symptoms may be aggravated over time, as observed in other situations.^5^^,^^30^ Also, the high prevalence of sleep disorders and their relationship with TMD, bruxism, and emotional factors deserves concern and may negatively impact the quality of life of DSs.

## Conclusion

In this study, dentists who were in quarantine had significantly more symptoms of depression, suggesting confinement may have a more negative impact on the lives of these professionals than the fact that they are actively working, despite the risks. The concern about Covid-19 was quite prevalent, being related to anxiety, stress, and poor sleep quality. Also, the high prevalence of sleep disorders and their relation to TMD, bruxism, and emotional factors deserve attention and can negatively affect the quality of life of the study population. Thus, further research is needed, especially in a post-pandemic scenario, since the symptoms may worsen over time.

## Appendix I Soelodemographlc Questionnaire

1. How old are you?
2. What is your Render?
  □ Male
  □ Female
3. What is your manta I status?
  □ Married/common-law marriage
  □ Single
  □ Divorced
  □ Widow/Widower
4. Do you have children who live with you and who are up to 14 years old?
  □ Yes
  □ No
5. What type of service do you work on?
  □ Public sector
  □ Private sector
  □ Public c Private
6. What state do you currently live in?
7. Do you belong to 8 risk eroup for Covid-19?
  □ Yes
  □ No
8. Are you worried about Covid-19?
  □ Extremely worried
  □ Very worried
  □ Worried
  □ Little worried
  □ Not worried
